# Evaluating open-note exams: Student perceptions and preparation methods in an undergraduate biology class

**DOI:** 10.1371/journal.pone.0273185

**Published:** 2022-08-18

**Authors:** Emily P. Driessen, Abby E. Beatty, Cissy J. Ballen

**Affiliations:** Department of Biological Sciences, Auburn University, Auburn, Alabama, United States of America; Central China Normal University, CHINA

## Abstract

Although closed-note exams have traditionally been used to evaluate students in undergraduate biology classes, open-note exams are becoming increasingly common, though little is known about how students prepare for these types of exams. We investigated student perceptions of and their preparation habits for online open-note exams in an undergraduate biology class, as compared to their previous experiences with closed-note exams in other classes. Specifically, we explored the following research questions: (1a) How do students perceive open-note exams impact their exam scores, their anxiety, the amount they studied, and the amount their peers studied? (1b) How do these perceptions impact performance outcomes? (2a) How do students prepare for open-note exams? (2b) How do these preparation methods impact performance outcomes? Results demonstrate students perceived increased exam scores, decreased exam-anxiety, decreased study time spent personally, and decreased study time spent by their peers for open-note exams, as compared to past experiences with closed-note exams. Open-ended survey responses analyzed through first- and second-cycle analyses showed students adapted their study habits by focusing on note preparation and broad conceptual understanding rather than rote memorization. Using linear mixed effects models to assess student performance, we found students who focused on understanding, note preparation and using external resources outperformed students who did not report those study habits. As institutions shift towards flexible and scalable assessments that can be used in face-to-face or online environments, the use of open-note exams can promote effective study habits and reward higher-order thinking with intentional guidance from the instructor.

## Introduction

Calls to action make specific recommendations for improving undergraduate biology education nationwide. For example, the American Association for the Advancement of Science (2011) outlined several core competencies intended to guide undergraduate biology education, including applying the process of science and understanding the relationship between science and society [1]. Consistently and notably absent from calls for change is the recommendation for students’ rote memorization of biology vocabulary or facts.

Nevertheless, one way in which student competencies are evaluated in higher education (i.e., Low-level Bloom’s Taxonomy closed-note multiple-choice question exams) rewards simple recall-based memorization of details rather than the development of the diverse skills needed to succeed in the scientific workforce [2–5] These types of closed-note exams are scalable, easily assessed, and perceived by some as more rigorous. However, open-note exams—those where students can consult textbooks, notes, or other class-related material during the exam—can be designed to be scalable and easily-assessed as well, and, *in addition*, open-note exams can reward a different skillset—one more aligned with recommendations for improving undergraduate biology education.

Contrary to closed-note exams, open-note exams render basic recall questions useless because students can simply refer to their notes. Rather, instructors may adapt open-note exams by developing assessments that evaluate higher-level skills such as conceptualization, problem solving, and reasoning [6]. For this reason, they have the potential to shift exam norms [7] as other disciplines have demonstrated [8]. However, the literature concerning open-note exams is mixed, producing both proponents and opponents of open-note exams.

Proponents of open-note exams propose a variety of benefits. First, proponents of open-note exams claim students learn how to gather and critically analyze material from multiple sources, as opposed to closed-note exams which reward short-term storage and quick retrieval [9–11]. Additionally, open-note exams decrease students’ exam anxiety [12–14] and minimize a desire to cheat [15, 16]. Green, Ferrante, and Heppard (2016) suggested that (1) higher education must evolve along with culture and technology, and (2) the ability to answer fact-based multiple-choice questions by rote memory is not adequately preparing students for future careers. Rather, instructional approaches that foster innovation, creativity, and independent thinking, such as open-note exams, train students for real-world operational decision-making [17].

Opponents of open-note exams propose several concerns. First, they suggest open-note exams lull students into a false sense of grade security, raising students’ *predictions* about their grades while not actually increasing student performance overall [18–20]. However, these claims have been contested, as other work shows open-note exams increased performance, as compared to closed-note exams [21, 22]. Jensen and Moore (2009) suggest that an inflated perception of one’s grades may encourage negative behaviors or study habits such as lower levels of class attendance and engagement, and less time spent reviewing class materials [19]. Moore & Jensen (2007) showed that compared to a class section who took closed-note exams, students assigned to a section that completed open-note exams were less likely to attend pre-exam help sessions, submit extra-credit assignments, and attend lecture [23].

Despite the mixed findings concerning open-note exams, research on open-note exams has continued, analyzing more nuanced scenarios. For example, Sato et al., (2015) explored the effect of open-note versus closed-note exams on student performance in the context of a biology classroom, paying particular attention to performance by Bloom’s levels. They found little difference in student performance on questions, regardless of their Bloom’s levels, between open- and closed-note exam takers. Sato et al. (2015) were particularly surprised that students who could use notes on Bloom’s 1 and 2 questions (i.e., memorization-based questions) did not outperform students who could not use notes. They concluded that to understand the relationship between open-note exams, performance, and learning, future research must focus on how students adapt their study habits to open-note exams [20].

Study habits include strategies students use to understand and retain class content. They also encompass how much time students spend studying and how students distribute their study time over the class of a semester [24, 25]. Because exam performance is highly correlated with study habits [26–29], the effectiveness of open-note exam taking may rely heavily on the specific exam preparation methods used by students. For this reason, we explored the impact of study habits for open-note exams on student performance. We focused on student performance because high-stakes exams often account for a sizeable proportion of students’ grades and can determine whether a student continues in STEM or leaves the field altogether [30].

Additionally, student perceptions of open-note exams may moderate student performance. For example, if students predict their grades will be higher simply by being allowed to use their notes on exams, then students may believe they do not need to study as much as they would for a closed-note exam [18–20]. This misled belief might impact preparation methods and consequently performance [25, 31]. For this reason, we correlated the amount and direction that students perceived their exam score would change for open-note exams compared to closed-note exams with performance. We also analyzed the relationship between the amount of time an individual studied for open-note exams compared to closed-note exams and performance, as well as the amount of time an individual perceived their peers studied for open-note exams. Another student perception we examined was test anxiety because of previous research demonstrating the strong impact of anxiety on student performance in college courses [32–41].

In this study, we examined study habits for and student perceptions of open-note exams longitudinally, over the course of one semester, surveying students after each of three mid-term open-note exams. Our longitudinal design is purposeful, given that previous literature shows student study habits change over time, after initial exposure to a new exam format. For example, Sato et al. (2015) examined whether the impact of open-note testing varied based on the extent of exposure to open-note exams, comparing those students who received open-note exams over the course of the entire quarter versus those with no prior open-note exam experience [20]. Findings demonstrated differences in both perceptions and performance between the two groups, suggesting that students adjust how they prepare for open-note exams after repeated exposure to the assessment format, highlighting the importance of a longitudinal examination of student perceptions and behaviors.

Open-note exams have surged in popularity alongside nation-wide increases in online classwork [42] and increased effort toward equitable and inclusive teaching strategies. However, to our knowledge, no study has addressed *how students study* differently for open-note exams compared to closed-note exams in undergraduate biology classes, if at all, and how different approaches relate to performance outcomes. Additionally, the correlation between student *perceptions of* open-note exams and student performance has yet to be examined.

To address these gaps in the literature, we investigated student perceptions of and their preparation habits for open-note exams in an undergraduate biology class, as compared to their previous experiences with closed-note exams in other classes. Specifically, we investigated the following research questions: (1a) How do students perceive open-note exams impact their exam scores, their anxiety, the amount they studied, and the amount their peers studied? (1b) How do these perceptions impact performance outcomes? (2a) According to open-ended responses, how do students prepare for open-note exams? (2b) How do these preparation methods impact performance outcomes? To address our research questions, we surveyed students after three open-note exams over a single semester, documented their perceptions and study habits, and analyzed how their responses related to performance outcomes.

## Methods

### Research design

This study used a mixed-methods approach, combining both qualitative and quantitative research methods and analyses. Our goal was to elucidate student perceptions of and study habits for open-note exams by collecting Likert scale (quantitative data) and open-response (qualitative data) survey data. We then related these two data types to student exam performance at three time points (i.e., Exam 1, Exam 2, and Exam 3) in the course using linear mixed-effects models. This research design allows us to explore the impact of (1) student perceptions of open-note exams on exam performance and (2) student study habits for open-note exams on exam performance. By using a repeated measures design (i.e., collecting survey responses after each of the three mid-term exams), we captured collective snapshots of our students’ perceptions of and study habits for open-note exams over the semester. Of note, Auburn University’s Institutional Review Board determined our research exempt and gave us written consent to proceed (protocol #20–603 EX 2012).

### Participants

Students in this study were enrolled in an online, introductory-level undergraduate biology course, Organismal Biology, taught during the spring 2021 semester by two of the authors (EPD & CJB). This course is the second in an introductory biology sequence, designed for those in biology-related majors to prepare them for future classwork. The study took place in a primarily white research-intensive university in the southeastern region of the United States.

### Course description

The Organismal Biology course is a three-credit class that includes two 75-min class sessions each week and is offered every semester. The main objective of the class is for students to develop an understanding of eukaryotic evolution, classification, structure, and the spectacular diversity of living organisms. In this class, students were randomly assigned to groups (ranging from 5 to 7 members) prior to the first class period, and these group assignments lasted for the duration of the semester. This class was online due to the COVID-19 pandemic, so students met in their groups in breakout rooms in Zoom during each class period. Each class period included lecture and group activities to reinforce the lecture material. For example, a typical class period may include some lecture, with an activity or iClicker question/group discussion every 10–15 min.

Summative assessment took place at four time-points (i.e., three mid-terms and a final exam). These exams were open-note, meaning students could use any of their notes—written or printed—on any of the exams. To make sure students weren’t using the internet or consulting with any outside help (e.g., group members, friends, family members), we enabled LockDown browser with Respondus monitor for each of the exams. Immediately after a student finished their exam, they could access their score. Each cumulative exam consisted of approximately 50 multiple-choice questions to be completed in two hours’ time. Most of the questions were application-based, asking students to interpret phylogenetic trees. Each student’s final grade consisted of the two best scores of the three mid-term exams, their final exam score, and participation points gained by answering in-class iClicker questions that mirrored application-based exam questions. We provide final grade information for transparency but note that we only used student exam scores for exam 1, 2, and 3, as individual measures of performance in this study.

### Data collection

After students completed each of their three mid-term exams, we encouraged them to take a voluntary post-exam Qualtrics survey. The survey instrument opened with an information letter detailing the purpose of the study to participants. Then, the survey prompted students to either consent or not consent before moving on to several survey questions. The remainder of the survey collected responses to four 5-point Likert scale questions and one open-response question (Table 1). Additionally, at the end of the semester, we downloaded student exam scores to use as a performance measure, so we could compare student study habits for each exam to their performance on that exam.

**Table 1:** The four 5-point Likert scale survey questions and answer choices used in this study.

Likert Scale Survey Items:	Response Type
1. Since I had the option to use notes on this exam, my score… 2. Since I had the option to use notes on this exam, my anxiety… 3. Since I was allowed to take this exam using my notes, I think the amount I studied… 4. Since students in our class were allowed to take this exam using our notes, I think the amount of time other students studied…	5-Point Likert Scale: (Greatly Decreased (1) to Greatly Increased (5))
**Open Response Survey Items**:	
1. How do you think you studied differently for this open-note exam compared to how you would study for a closed-note exam?	Short Response

### Data analysis

We downloaded survey responses one week after the end of the semester. First, we removed all student data from students who did not consent to be a part of the study. Given that we used many different analytical methods to answer our research questions, we detail each part of the analysis of the Likert scale responses and the open-ended responses in two sections, starting with “Likert Scale Response Analysis”. We conducted all analyses and produced all plots for this research in R version 4.0.3 [43]. We edited plots in BioRender.com.

#### Likert scale response analysis—Student perceptions

*Student perceptions*: *Descriptive analysis*. We calculated response rates for the four Likert scale questions and the open-ended survey question—*How do you think you studied differently for this open-note exam compared to how you would study for a closed-note exam*?—by dividing the total number of consenting responses by the total number of students enrolled in the course. Next, we assessed Likert scale student responses using a Likert package in R [44], calculating the proportion of students reporting a range of responses from “greatly decreased” to “greatly increased” to each of the prompts. We visualized these findings using the plot function.

*Student perceptions*: *Linear mixed-effect model analysis*. We used the nlme package [45] to create linear mixed-effects models, examining the impact of student perceptions of open-note exams on student performance. We first created a model including all four student perceptions measured as our fixed effects (i.e., how the ability to use notes on their exams would impact their exam performance, anxiety levels, the amount they studied, and the amount they thought their peers studied), treating the Likert scale responses as categorical variables. Then, because individual students were sampled longitudinally over the study period, we used a repeated measures design to account for longitudinal measures from a single student by including Student ID as a random effect. After running our first model, we removed one fixed-effect at a time, choosing to remove the fixed-effect with the least significant p-value for each subsequent model. We selected the best fit model using Akaike’s Information Criterion (AIC; [46]), selecting the model with the lowest AIC. In the results section, we included only the relationships that returned significant results, based on a p-value less than 0.05 and confidence intervals that exclude zero.

Next, we created a separate model for each of the four student perceptions measured (i.e., how the ability to use notes on their exams would impact their exam performance, anxiety levels, the amount they studied, and the amount they thought their peers studied), so we could conduct Tukey based post-hoc analysis functions to obtain pairwise significance between exam scores using the emmeans package [47]. Then, to visualize all statistically significant relationships, we created customized plots (i.e., combined violin, scatter, and boxplots) in the ggplot2 package [48].

#### Open-ended response analysis—Student study habits

*Student study habits*: *Descriptive analysis*. First, we calculated response rate for the open-ended survey question—*How do you think you studied differently for this open-note exam compared to how you would study for a closed-note exam*?—by dividing the total number of consenting responses by the total number of students enrolled in the course.

**Fig 1:**
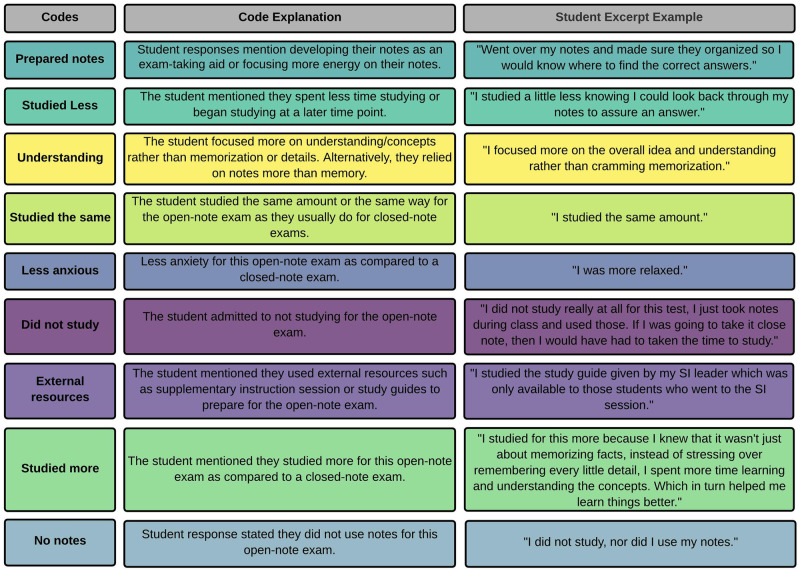
Emergent codes in student open responses to the open-ended question “*How do you think you studied differently for this open-note exam compared to how you would study for a closed-note exam*?*”*

Next, two of the authors (AEB & EPD) individually reviewed all the students’ responses to the open-ended question and generated codes using inductive coding [49]. We also took detailed analytic notes at that time [50]. We convened to compare codes and develop one unified coding rubric. Using the unified rubric, AEB and EPD coded a set of 40 responses individually. We met together to compare codes and revise the rubric. We used constant comparison methods to ensure quotes within a category were not too different from each other to warrant the creation of a new theme [51]. This process was repeated until we were confident with the rubric, resulting in the following codes: (a) prepared notes, (b) studied less, (c) understanding, (d) studied the same, (e) less anxious, (f) did not study, (g) external resources, (h) studied more, and (i) no notes (Fig 1).

Once the final rubric was established, AEB and EPD individually used the rubric to code the entire data set. Each code is mutually exclusive, meaning an excerpt of text could only be coded as one code. However, students’ full responses could include multiple themes. We reached an initial 77.7% agreement, and then met to code to consensus. After we reached consensus, we calculated percentages for each code by dividing the total number of responses assigned for each code by the total number of student responses. After calculating these percentages, we visualized the frequency of each code by constructing bar plots in the ggplot2 package [48], creating one plot for the codes overall (the average frequency of the code over all three surveys) and one plot for the codes as broken down by each survey timepoint (i.e., exam 1, exam 2, and exam 3). For the overall plot, we included frequencies for all codes, however, for the plot broken down by survey timepoint, we only included information for the codes that demonstrated visual differences between timepoints.

*Student study habits*: *Linear mixed-effect model analysis*. To examine the impact of student study habits for open-note exams on student performance, we first reformatted thematic codes as binary data. This means if a student mentioned a particular code in their open-ended response, then they would receive a 1 (yes) for that code. However, if they did not mention a particular code in their open-ended response, then they would receive a 0 (no) for that code. For example, if a student mentioned the code “focusing on understanding” then they were assigned a 1 for that variable. We assigned these values for each qualitative code in the dataset.

After we reformatted the qualitative data into binary data, we created a linear mixed-effects model using the nlme package [45] that included all nine student study habit codes as our fixed-effects (i.e., prepared notes, studied less, understanding, studied the same, less anxious, did not study, external resources, studied more, and no notes; Fig 1). Then, because individual students were sampled longitudinally over the study period, we used a repeated measures design to account for repeated sampling from a single student during longitudinal measures by including Student ID as a random effect. After running our first model, we removed one fixed-effect at a time, choosing to remove the fixed-effect with the least significant p-value for each subsequent model. We selected the best fit model using AIC [46]. In the results section, we only included the study habits that returned significant results. We determined statistical significance and graphically depicted the data as previously described.

## Results

### Likert scale response results—Student perceptions

#### Student perceptions: Descriptive results

**Fig 2:**
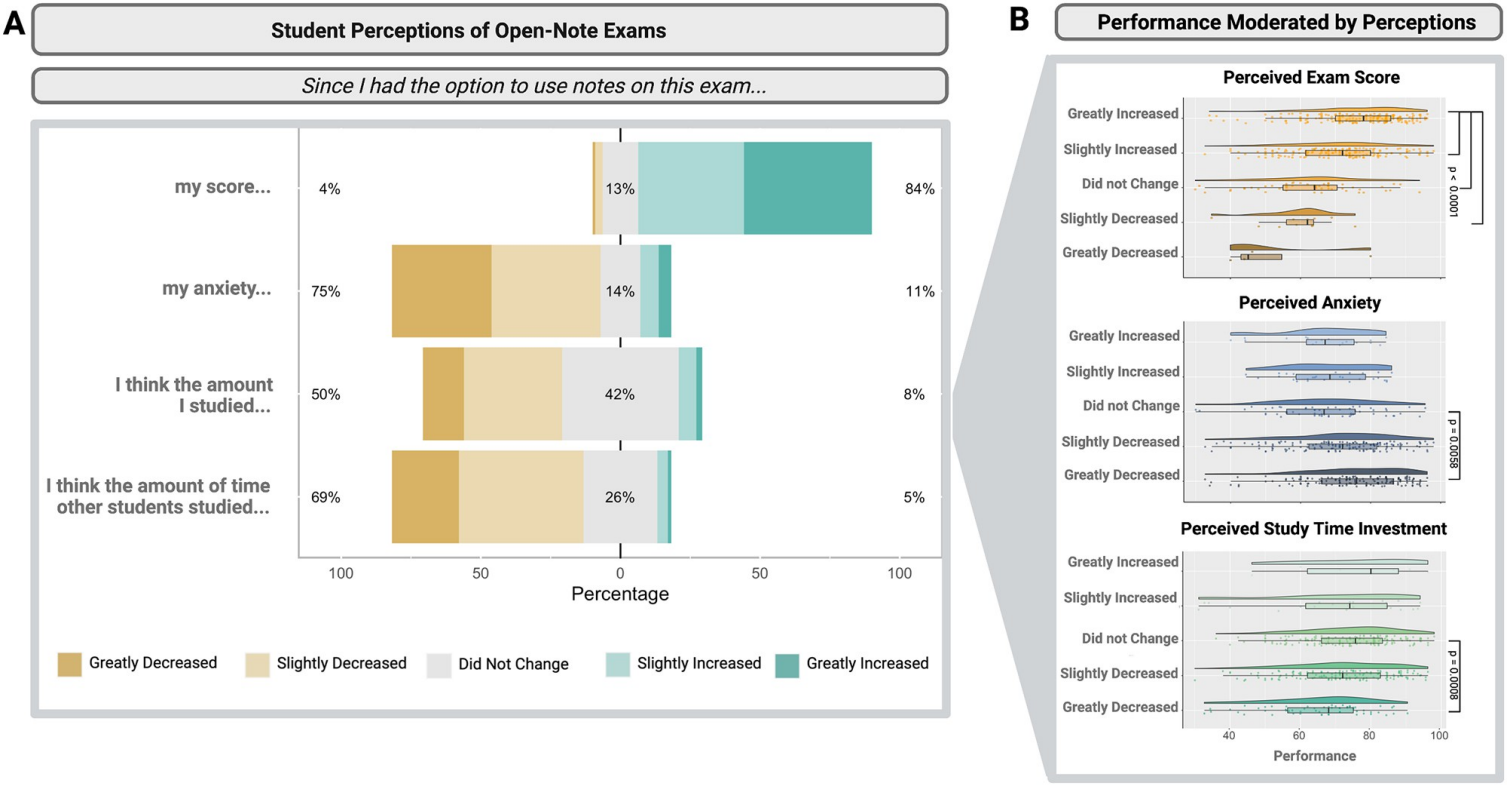
Student perceptions of open-note exams. Students were asked a series of 5-point Likert scale questions. (A) Students report perceptions of increased exam performance, decreased anxiety, decreased personal study time investment, and decreased peer study time investment on open-note exams as compared to closed-note exams. (B) Only students reporting that the ability to use notes on their exam “greatly increased” their exam score, “greatly decreased” their anxiety, or “greatly decreased” their study investment had significant impacts on performance.

For the four Likert scale questions (Table 1), we received 445 complete responses (i.e., 157 responses after exam 1 [157/218; 72.0% response rate], 147 responses after exam 2 [147/218; 67.43% response rate], and 105 responses after exam 3 [105/218; 48.17% response rate]). After calculating the response rates, we calculated the proportion of students reporting a range of responses from “greatly decreased” to “greatly increased” to each of the prompts. Results demonstrate that students generally equate open-note exams with increased exam scores, decreased anxiety, and decreased studying (Fig 2A).

#### Student perceptions: Linear mixed-effect model results

Next, we examined the relationship between student perceptions of open-note exams and exam performance using linear models. The best fit model for examining the relationship between student perceptions of open-note exams and student performance on those exams included all four student perceptions (i.e., how the ability to use notes on their exams would impact their exam performance, anxiety levels, the amount they studied, and the amount they thought their peers studied) as fixed effects and Student ID as a random effect. Using this model, three of the Likert scale perceptions displayed clear relationships with student performance: perceived exam score (F_(1,202)_ = 13.78, p < 0.0001); anxiety (F_(1,202)_ = 4.62, p = 0.0005); and study time (F_(1,202)_ = 4.63, p = 0.0005; Fig 2B). We then ran a model for each individual perception as a fixed effect with Student ID as a random effect, so we could use these models to conduct Tukey based post-hoc analysis functions. Ultimately, this allowed us to find any differences between Likert scale categories for each individual perception.

We found (1) that students who reported the ability to use notes on their exam “greatly increased” their exam score performed at a significantly higher level than those who claimed their score “slightly increased (7.60 ± 1.37 (SE), p < 0.0001), “did not change” (12.79 ± 1.95 (SE), p < 0.0001), or “slightly decreased” (18.25 ± 3.55 (SE), p < 0.0001); (2) students who reported “greatly decreased” anxiety performed at a significantly higher level than students reporting no change (7.45 ± 2.15 (SE), p = 0.0058); and (3) students who reported “greatly decreased” study time investment performed at a significantly lower level than those who reported no change (-8.81 ± 2.15 (SE), p = 0.0008). Other incremental comparisons had no significant effect on student performance (Fig 2B). We did not observe differences between student responses to the Likert scale questions over time (p > 0.30 in each case).

### Open-ended response results—Student study habits

#### Student study habits: Descriptive results

For the open-ended survey question—*How do you think you studied differently for this open-note exam compared to how you would study for a closed-note exam*?—we received 347 total responses (i.e., 140 responses after exam 1 [140/218; 64.22% response rate], 135 responses after exam 2 [135/218; 61.93% response rate], and 72 responses after exam 3 [72/218; 33.03% response rate]). However, we excluded 65 responses (18.68%) from our analysis because they did not fit our codes due to difficulty of interpretation or ambiguity. This left us with 107 coded responses for exam 1, 114 coded responses for exam 2, and 61 coded responses for exam 3.

**Fig 3:**
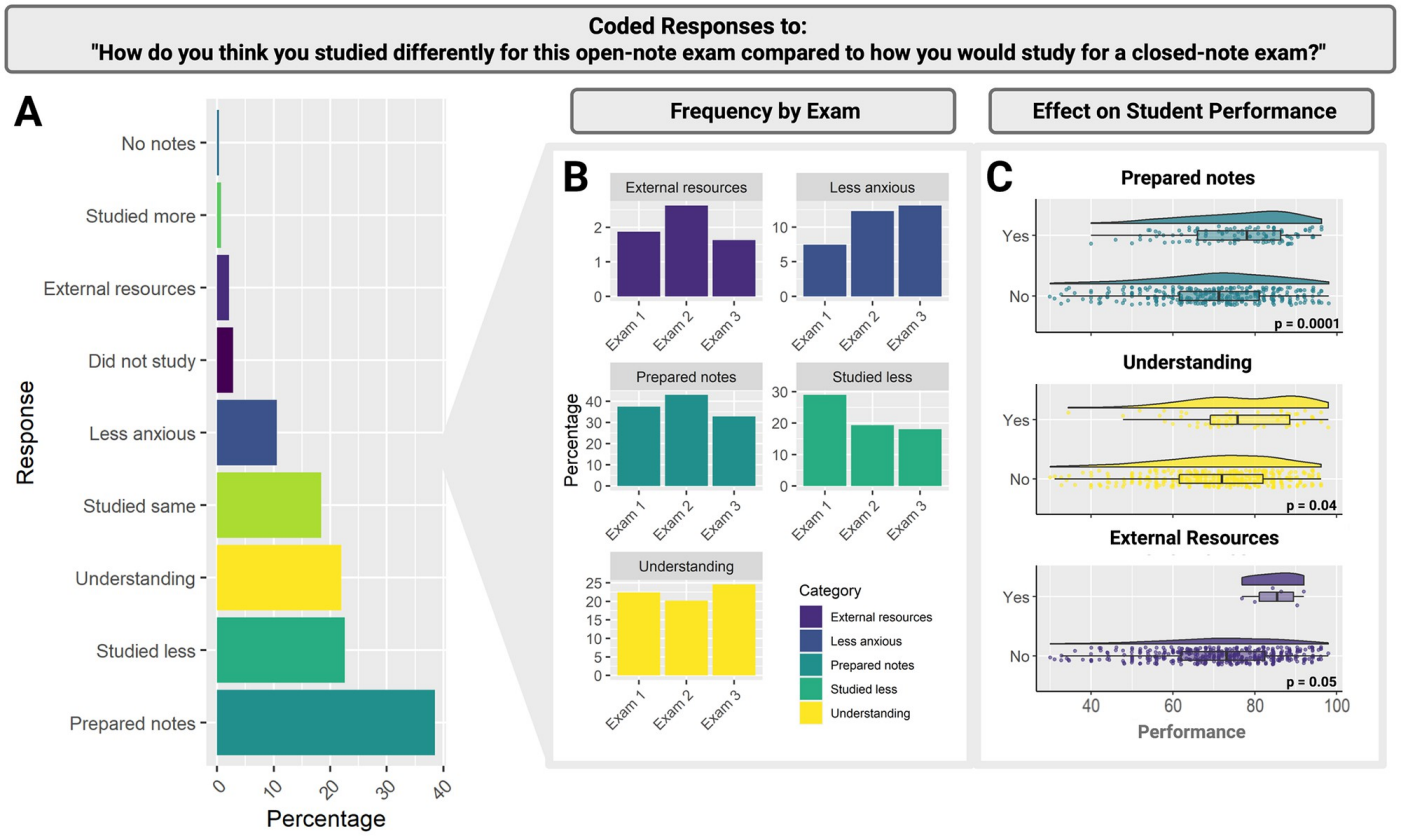
Adapting study habits to open-note format. Students were asked to respond to the following prompt: “*How do you think you studied differently for this open-note exam compared to how you would study for a closed-note exam*?*”* (A) Student responses are ordered by frequency of student reporting. (B) Longitudinal plotting of student responses indicate student approaches to open-note exams may vary over time in terms of students’ use of “External Resources”, feeling “Less Anxious”, having “Prepared Notes,” having “Studied Less” and focus on “Understanding”. (C) Student performance increased in response to study habit adaptations that include having “Prepared Notes,” an increased focus on “Understanding,” and the use of “External Resources”.

We identified three important coded responses to the question “*How do you think you studied differently for this open-note exam compared to how you would study for a closed-note exam*?*”* Over the three exams, students commonly reported they “prepared notes” for open-note exams. That is, roughly 40% of students mentioned developing their notes as an exam-taking aid or focusing more energy on their notes for these open-note exams than they would have for a closed-note exam (Fig 3A). Another common study strategy for open-note exams was to focus on “understanding” the material rather than memorization (21.91%; Fig 3A). Lastly, students mentioned accessing “external resources” for studying (2.12%; Fig 3A). In our study, we use the term external resources as a catchall term for attending supplemental instruction sessions and using study guides.

When we analyzed student responses longitudinally by exam (i.e., exam 1, 2, or 3), only the following five codes varied by exam: (1) external resources, (2) less anxious, (3) prepared notes, (4) studied less, and (5) understanding (Fig 3B). While many of these relationships changed over time, the only general trend we found was that, as time went on, students were less likely to report “studying less”, which may indicate adaptation to the realities of needing to study for the open-note exam format. Although it should be noted that when students were asked to report their study investment through Likert scale options, their responses did not vary significantly over time. So, while students were less likely to report studying less through short answer responses, we did not identify any variation over time based on quantitative Likert data. Finally, while students often reported “prepared notes” as an adaptation to open-note exams, the frequency of this response decreased for the third exam (Fig 3B).

#### Student study habits: Linear mixed-effect model results

The best fit model for explaining the relationship between student study habits for open-note exams and student performance on those exams included all nine study habit codes (i.e., prepared notes, studied less, understanding, studied the same, less anxious, did not study, external resources, studied more, and no notes) as fixed effects and Student ID as a random effect. Students who focused on preparing notes for open-note exams outperformed students who did not approach studying this way (F_(1,229)_ = 14.93, p = 0.0001; Fig 3C). Students who focused on understanding to prepare for open-note exams outperformed students who did not approach studying this way (F_(1,229)_ = 4.34, p = 0.04; Fig 3C). Lastly, students accessing “external resources” for studying outperformed those who did not report this (F_(1,229)_ = 3.85, p = 0.05; Fig 3C).

## Discussion

We assessed how students perceived open-note exams compared to closed-note exams, whether and how their study habits changed compared to closed-note exams, and how each of these factors impacted student performance. In the following section, we place our findings in the context of current research.

### Student perceptions of open-note exams

Student perceptions of their performance on open-note exams may be consistent with previous literature. For example, Jensen and Moore (2009) showed open-note exams contributed to an overinflated confidence in performance [19], and this finding may align with our finding that an overwhelming majority of students in our study thought the ability to use their notes would increase their exam score. However, those students who thought the ability to use notes on their exams would “greatly increase” their exam performance significantly outperformed students who thought that same ability would either only “slightly increase,” “not change,” or “slightly decrease” their performance. This may demonstrate students who really thought they would benefit from the exams really did benefit in terms of better performance, potentially demonstrating that those students did not have an overinflated confidence in performance. However, this warrants further investigation, especially because students were able to see their actual score on the exam prior to taking the survey asking about their perceived performance. Additionally, students reported decreased test anxiety levels, which also aligns with previous literature [12–14, 52].

Our finding that students perceived they studied less for their open-note exams, as compared to closed-note exams, is rarely discussed in literature, and could be interpreted in several ways. The most straight-forward interpretation is that students studied the same way for open-note exams as they would for closed-note exams, but for less time. However, students likely studied differently for open-note exams, especially those that test at higher Blooms’ taxonomy levels (e.g., application, analysis, and evaluation; [53]), and this may have contributed to their perceptions of time spent preparing. For example, students commonly prepared organized notes that were easy to navigate during the exam, relying on those notes as a life raft during the exam. Students who reported studying less for open-note exams may hold a different interpretation of studying, discounting the preparation of notes as a form of studying. Open-note exams alternatively may promote better study habits [54] given that preparation of notes can be an active method of studying, improving student performance [55, 56]. Active methods of studying are more effective than passively memorizing facts by re-reading the textbook or study materials, which is time consuming and does not lead to meaningful learning outcomes [55], particularly those outlined in the Vision and Change recommendations [1]. While our results cannot explain why students reported studying less, our future work will explore these interpretations of exam preparation.

Three of the Likert scale findings showed relationships with performance outcomes: first, students who reported “greatly increased” perceived exam scores performed at a significantly higher level than those who claimed their score “slightly increased,” “did not change,” or “slightly decreased;” second, students who reported “greatly decreased” anxiety performed at a significantly higher level than students reporting no change; and third, students who reported the amount of time they invested into studying “greatly decreased” performed at a significantly lower level than those who reported no change. Positive performance outcomes were only associated with students who selected the maximum Likert scale responses (i.e., greatly decreased anxiety and study time investment or greatly increased exam score). For example, greatly decreased anxiety positively impacted student performance, but there was no impact of increased or slightly decreased anxiety. Similarly, greatly decreased study time investment negatively impacted student performance, but there was no impact of increased or slightly decreased study time investment on performance. To conclude that student responses to a Likert scale reflect incremental differences in student affect might not fully capture the complex relationships between affect, behavior, and cognition. For example, previous work found a moderate amount of anxiety heightened focus and alertness during assessments, but too much anxiety impaired performance by diverting cognitive resources away from the exam [57]. Similarly, we may only see performance effects for students reporting more ‘extreme’ Likert responses, but not among those with milder reports.

Our finding that student perceptions did not significantly shift as experience with open-note exams increased shows that students are not likely to adapt their perceptions of the amount they need to study for an open-note exam as compared to a closed-note exam. Our finding that students’ reported anxiety did not change over time suggests that open-note exams can keep student’s perceived test anxiety lower than closed-note exams, even after repeated exposure.

### Student study habits for open-note exams

We found that students who focused on note preparation outperformed their peers, aligning with previous research showing that learning is enhanced by going through the motions of organizing and preparing notes [58]. Note preparation serves as an active and effective form of exam preparation. These findings support previous recommendations for instructors to model active study strategies during class, such as developing structured and accessible notes [25]. Our results highlight the importance of modelling the study habits that are effective for this exam type.

Students also focused on “understanding” the material rather than memorization for open-note exams. Students may have written this because they could reference their notes for detail, so they did not need to memorize that information. Rather, they might focus on higher-order concepts, applying the course content to different scenarios. However, students may have been responding to the expectations of the assessment, which, in this research, were different because the exams were open-note. Specifically, the instructors shifted away from a ‘fill in the blank’ style question that students could easily find in their notes. In other words, both students and the instructors may make changes with the knowledge that the exams are open-note: the students in their studying behaviors, and the instructors in how they constructed exam questions. Future research can explore how instructors write open-note exam questions, and how they differ or are similar to closed-note exams.

Students’ focus on understanding broad concepts rather than memorization supports previous reports showing students prepare for open-note exams by gathering and critically analyzing material from multiple sources as opposed to focusing on storing information for quick retrieval in preparation for a closed-note exam [11]. Additionally, it lines up with literature showing students expect open-note exams will emphasize understanding and analysis [8]. We also found that students who reported focusing on “understanding” to prepare for open-note exams outperformed students who did not report studying this way. These findings, although exploratory, are perhaps the most convincing pieces of evidence we have that open-note exams have the potential to move the goal of undergraduate biology assessments away from memorization and toward deeper understanding and application.

Lastly, we found that students who used “external resources,” such as attending supplemental instruction sessions and using study guides, outperformed those who did not report preparing this way. Previous research shows supplemental instruction, in which a senior student facilitates learning for undergraduate peers in a challenging class, leads to higher grades, lower failure and withdrawal rates, and higher retention and graduation rates [59, 60]. The supplemental instructor in this study, in collaboration with the instructors, developed study guides that outlined the important topics students were expected to know for the exam and included practice questions for students. The supplemental instruction sessions consisted of careful review of the study guide and associated materials, as well as a question-and-answer session.

Our analysis of student responses longitudinally by exam (i.e., exam 1, 2, or 3) showed students reported preparing notes less for the third exam (Fig 3B). We hypothesize this decrease could be due to either (1) time demands on the students, given the third mid-term occurred two weeks before final examination week, and students enrolled in our class are often enrolled in many other challenging pre-med classes, or (2) as part of the design of our class, we allowed students to drop one of the mid-term exams, so if they were already doing well in the class, then they may have used the third exam as their drop exam and therefore not studied for it at all, much less with note preparation in mind. These hypotheses, as well as any alternatives, should be tested to delve into the reasoning behind changing study strategies for open-note examinations.

### Implications for practice

We offer several recommendations for undergraduate biology instructors who are considering incorporating open-note examinations into their classrooms. First, we encourage post-secondary biology instructors to ask themselves one key question: what do I want students to gain from their learning and exam experiences? If the objective is for students to focus on understanding rather than memorizing course materials, writing assessment questions that reflect this objective will encourage students to think deeply about the material and reward those who do. Students may benefit from their instructor explaining how their open-note exams are designed to encourage them to specifically focus on understanding, along with the study habits that have previously been shown to increase student performance and learning (e.g., synthesizing notes, focusing on understanding, self-quizzing [25]). Second, we encourage biology education researchers to evaluate how open-note exams are commonly designed and implemented in post-secondary biology classrooms, paying special attention to the Bloom’s taxonomy level of each exam question, whether the instructor explains the difference between an open-note and a closed-note exam, and whether the instructor models evidence-based study habits for their students. This will advance our knowledge on the most effective forms of open-note exams, and address why there are mixed-findings in the literature concerning their efficacy.

## Limitations

There are several limitations to the current study. First, we conducted this work amid a global pandemic, which disrupted the lives of students and impacted how they experienced their courses. Students were required to enroll in the remote, online course because of institutional health and safety concerns rather than preference. This means that the student study population, who were taking online, open-note exams, may not represent students who would normally opt for this type of course, potentially impacting our results. Relatedly, the generalizability of these findings is limited because the study took place at a selective, primarily white southeastern institution. Our work in the future will expand beyond this setting, one that is commonly overrepresented in research [61], and address questions related to open-note exams across community colleges, minority-serving institutions, primarily undergraduate institutions, and other student populations. Another limitation includes the fact that the survey we used in our analyses represented single-item survey responses rather than survey constructs. Survey constructs, which are composed of several single-item responses, are preferred because they can be tested for validity by analyzing whether participants answered similarly. Finally, it is important to note that this work examines student perceptions of and study habits for open-note exams that consisted of multiple-choice questions. We acknowledge the importance of work examining perceptions of and study habits for open-note exams consisting of different question-types (e.g., short answer, essay, and design questions), and we note it may produce different results.

## Conclusions

The perceptions students have, and the approaches students take to prepare for an exam can affect performance and (potentially) learning, necessitating the investigation of student exam perceptions and exam preparation relevant to the exam type. However, investigations such as these have yet to be conducted with novel types of exams such as open-note exams. Our research on one such novel exam, open-note exams, indicates that (1) students perceived they would perform better on open-note exams while studying less and experiencing less anxiety, (2) study habits including focusing on note-preparation, deep understanding, and application may lead to higher performance in biology classes that use open-note exams, (3) students often practice unsuccessful study habits, so they may benefit from instructor guidance on effective preparation techniques, and (4) in order to effectively assess open-note exams, future research study designs must take into account the appropriateness of the exam design. We emphasize that low-level Bloom’s taxonomy, closed-note, multiple choice question exams do not effectively address Vision and Change needs for student success. However, when properly designed, open-note exams can promote in-depth, higher-level thinking, as well as effective study habits.
